# Limits to the usability of iconic memory

**DOI:** 10.3389/fpsyg.2014.00971

**Published:** 2014-08-29

**Authors:** Ronald A. Rensink

**Affiliations:** Department of Psychology and Department of Computer Science, University of British ColumbiaVancouver, BC, Canada

**Keywords:** iconic memory, feedback connections, visual search, visual attention, visual memory

## Abstract

Human vision briefly retains a trace of a stimulus after it disappears. This trace—iconic memory—is often believed to be a surrogate for the original stimulus, a representational structure that can be used as if the original stimulus were still present. To investigate its nature, a flicker-search paradigm was developed that relied upon a full scan (rather than partial report) of its contents. Results show that for visual search it can indeed act as a surrogate, with little cost for alternating between visible and iconic representations. However, the duration over which it can be used depends on the type of task: some tasks can use iconic memory for at least 240 ms, others for only about 190 ms, while others for no more than about 120 ms. The existence of these different limits suggests that iconic memory may have multiple layers, each corresponding to a particular level of the visual hierarchy. In this view, the inability to use a layer of iconic memory may reflect an inability to maintain feedback connections to the corresponding representation.

## INTRODUCTION

It has long been known that human vision retains a brief trace of any stimulus it encounters (see e.g., Loftus and Irwin, 1998). This trace, often referred to as *iconic memory*, has been a focus of investigation for several decades (e.g., Sperling, 1960; Coltheart, 1980; Ruff et al., 2007; Sligte et al., 2010). It is sometimes considered to be a “visual echo” that can act as a surrogate, i.e., that as long as it lasts, its contents can be used in much the same way as if the stimulus were still visible. But there is little consensus as to what—if any—function iconic memory may have (see e.g., Pashler, 1998). On one hand, it has sometimes been considered a simple side effect, with potentially deleterious effects on perception (Haber, 1983). On the other, it could potentially increase the amount of information that could be extracted from a brief presentation (Haber, 1971).

Iconic memory has most often been studied via *partial report*, in which observers are briefly shown an array of a dozen or so items and then asked to report a subset that is cued after the array disappears (Sperling, 1960; Averbach and Coriell, 1961). Various studies have also examined the extent to which iconic representations can be used in memorization and recognition tasks (e.g., Loftus et al., 1992; Keysers et al., 2005) as well as change detection (e.g., Becker et al., 2000; Sligte et al., 2010). All assume that iconic memory is equally available to any visual process. But is this really so? Or might it be used to different extents by different processes?

**FIGURE 1:**
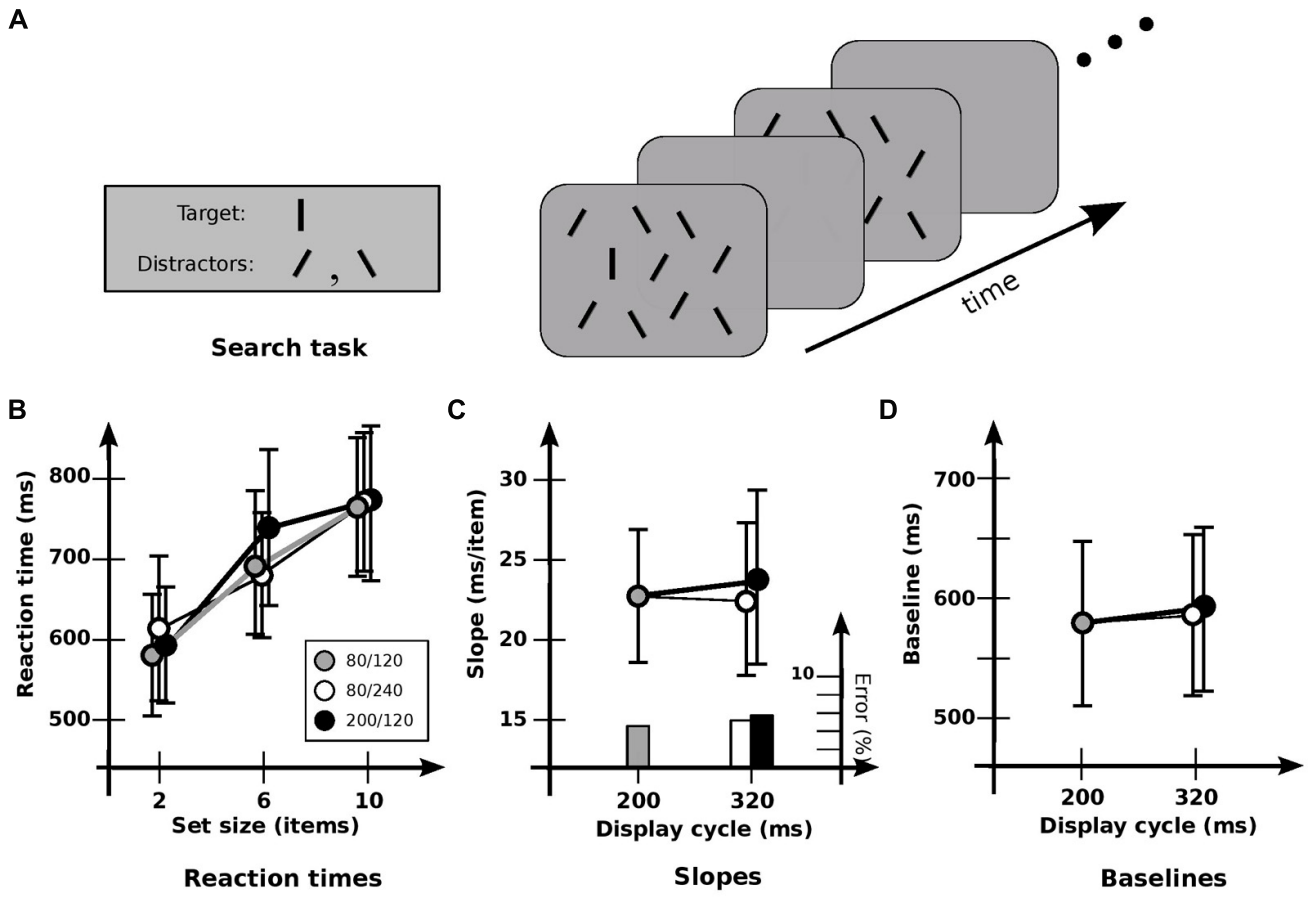
**Experiment 1A: detection of orientation. (A)** General setup. Target is a vertical line; distractors are lines tilted ± 30°. Displays “flickered” until subject responded, or 5 s elapsed. **(B)** Response times and error rates as a function of set size for the three cadences. **(C)** Data recast as slopes. Slope for base cadence (23.0 ms/item) is unaffected by either an increase in off-time (22.1 ms/item) or an increase in on-time (24.4 ms/item). Note that since these are target-present slopes from a presumably self-terminating search, the search speed itself is obtained by multiplying by a factor of about 2. The resultant speeds are about 50 ms/item, similar to those found elsewhere. **(D)** Data recast as baselines. Values for the base cadence (564 ms) are not significantly affected by an increase in off-time (576 ms) or on-time (580 ms). Error bars indicate standard error of the mean.

To investigate this, a *flicker search* paradigm was developed (**Figure 1**). This is a variant of visual search, where the observer must determine as quickly as possible the presence or absence of a given *target* among a set of non-target items (or *distractors*) in a display; different visual operations can be tested by different choices of target and distractors (e.g., Treisman and Gormican, 1988; Wolfe and Horowitz, 2004). In flicker search, observers search displays that are visible only intermittently: after a fixed time (the display duration, or *on-time*), the display is blanked for some fixed interval [the interstimulus interval (ISI), or *off-time*], this cycle then repeated until the observer responds or times out. (To enable maximal use of iconic memory, no masks are present.) For many kinds of search task, the time needed to respond is proportional to the *set size* (the number of items in the display), likely reflecting the application of an attentional mechanism (Treisman and Gormican, 1988; Wolfe and Horowitz, 2004). If this mechanism is sufficiently slow, search will require the scan of the blank intervals^1^. The question then is whether the speed of search through a blank interval (i.e., iconic memory) is the same as through the representation that gave rise to it. This can be answered by comparing performance when iconic memory is used for different fractions of the display cycle.

Such a “full scan” technique removes several potential problems of partial report, such as complications due to memory consolidation and transfer; it also reduces the likelihood of observers using different strategies (cf. Estes and Taylor, 1964). Consequently, it may provide a more precise estimate of iconic properties. Importantly for the issue at hand, it also allows a wide variety of tasks to be examined using the same general framework.

In what follows, it will be shown that this approach can indeed work, and provides converging evidence that iconic memory can act as a surrogate for a stimulus that has suddenly disappeared. But it will also be shown that iconic memory is available to different tasks for different amounts of time, with these limits clustering into a few groups, each likely corresponding to a particular level of the visual hierarchy. As such, it will be argued that this approach can shed considerable light on the nature of the various levels of the visual hierarchy, and on the nature of the feedforward and feedback^2^ connections between them.

## GENERAL METHOD

Unless otherwise specified, each experimental condition used three timing patterns, or *cadences*: a base cadence of 80/120 (80 ms on; 120 ms off), and two longer cadences of 80/240 and 200/120, created by increasing the off- and on-times respectively by 120 ms. Each condition tested 12 observers, with order of cadence counterbalanced. Observers were seated 57 cm from the monitor. Displays subtended 11.5° × 8.5° in visual angle, and contained 2, 6, or 10 items, with spacing controlled to keep item density constant. For detection conditions, the target was present on a randomly selected half of trials; otherwise, the target was always present in each display. Items were ~1° in extent, the exact size depending on the condition tested.

Lighting level was sufficient to allow color to be easily seen (i.e., above the mesopic range). A cathode-ray tube (CRT) display was used for all conditions. Blank fields and display backgrounds were both medium gray, resulting in a continual flickering of the items on a static background. All items were black, apart from those in the contrast polarity condition. The appearance of a gray field after the disappearance of an item therefore corresponded to an increase rather than a decrease of phosphor activation, ensuring that phosphor persistence could not significantly affect the results.

All experimental conditions were run on a Macintosh computer using VSearch software (Enns et al., 1990). Observers were instructed to maintain fixation during each trial, to detect the target as quickly as possible, and to keep error rates below 5%. Responses were given via one of two response keys. All observers completed four sets of 60 trials in each condition. Performance was measured in terms of reaction times (RTs) that were averaged for each observer; these were then recast into search speed (average target-present slope^3^) and baseline (estimated time needed for a single item in the display). A trial timed out—and was considered an incorrect response—if more than 5 s was needed.

## EXPERIMENT 1

This experiment examined whether iconic memory can support visual search for a simple feature. The target was a black vertical line 0.8° long; distractors (non-targets) were similar lines oriented ± 30° to the vertical (**Figure 1A**). Observers were asked to detect the presence or absence of the target.

Condition 1A examined detection for the three cadences of 80/120, 200/120, and 80/240. Search of this kind typically has target-present slopes of 15–30 ms/item in a static display (cf. Treisman and Gormican, 1988). Search here was similar: RTs showed a strong effect of set size [*F*(2,10) = 22.8; *p* < 0.0001], with an average slope of 23.2 ms/item (**Figure 1B**). However, no significant effect of cadence was found [*F*(2,10) = 0.711; *p* > 0.5], nor any significant interaction between set size and cadence [*F*(4,10) = 1.29; *p* > 0.3]. Cadence had no significant effect on either slopes [*F*(2,10) = 0.151; *p* > 0.8; **Figure 1C**], or baselines [*F*(2,10) = 0.47; *p* > 0.6; **Figure 1D**]. Error rates were much the same for all cadences, indicating that no speed-accuracy trade-offs occurred. As such, these results indicate that the information in iconic memory can survive without serious degradation for at least 240 ms, consistent with conclusions obtained elsewhere (e.g., Sperling, 1960; Graziano and Sigman, 2008). And the lack of effect of different cadences—essentially, different switching rates—indicates little cost of switching between visual and iconic representations.

As a test of whether the memory being used actually is iconic memory, Condition 1B compared performance for the 80/240 cadence against two others: a 80/0 cadence (i.e., a display that remained on), and a 80/320 cadence (in which the blank interval was 320 ms). Paired *t*-tests showed that slopes and baselines for 80/240 and 80/0 conditions were virtually identical (*p* > 0.9 and *p* > 0.5, respectively), both with a slope of 20.5 ms, indicating that the flicker had little effect. Extending the blank duration to 320 ms showed a similar lack of effect (*p* > 0.2 and *p* > 0.9, respectively). However, slopes for the 80/240 and 80/320 conditions were 20.5 and 25.2 ms/item respectively, suggesting a slight degradation for the longer blank; indeed, a more detailed analysis^4^ indicates that performance is a function of on-time plus a *usable duration* (*u)* of 246 ± 57 ms.

Taken together, these results are consistent with other findings showing that the information in iconic memory can survive without serious degradation for several 100 ms (e.g., Sperling, 1960; Graziano and Sigman, 2008). The speed of search was much the same throughout, not only supporting the proposal that attentional selection and iconic memory involve common representations (Ruff et al., 2007), but indicating that the iconic representation can be used as easily and effectively as the one used in “regular” vision, with the switch between visible and iconic representations requiring little or no time.

## EXPERIMENT 2

To examine the extent to which iconic memory can be used for other tasks, Experiment 2 examined its involvement in change detection. Based on the difficulty of detecting change in the absence of attention (i.e., change blindness), it has been proposed that most unattended structure is detailed but volatile, with iconic memory being the quickly dissipating remnant of this representation after the stimulus disappears (Rensink et al., 1997; Rensink, 2000a). Subsequent work (Becker et al., 2000) supported this proposal, indicating that the cueing of iconic memory can guide attention, and thereby facilitate change detection.

**FIGURE 2:**
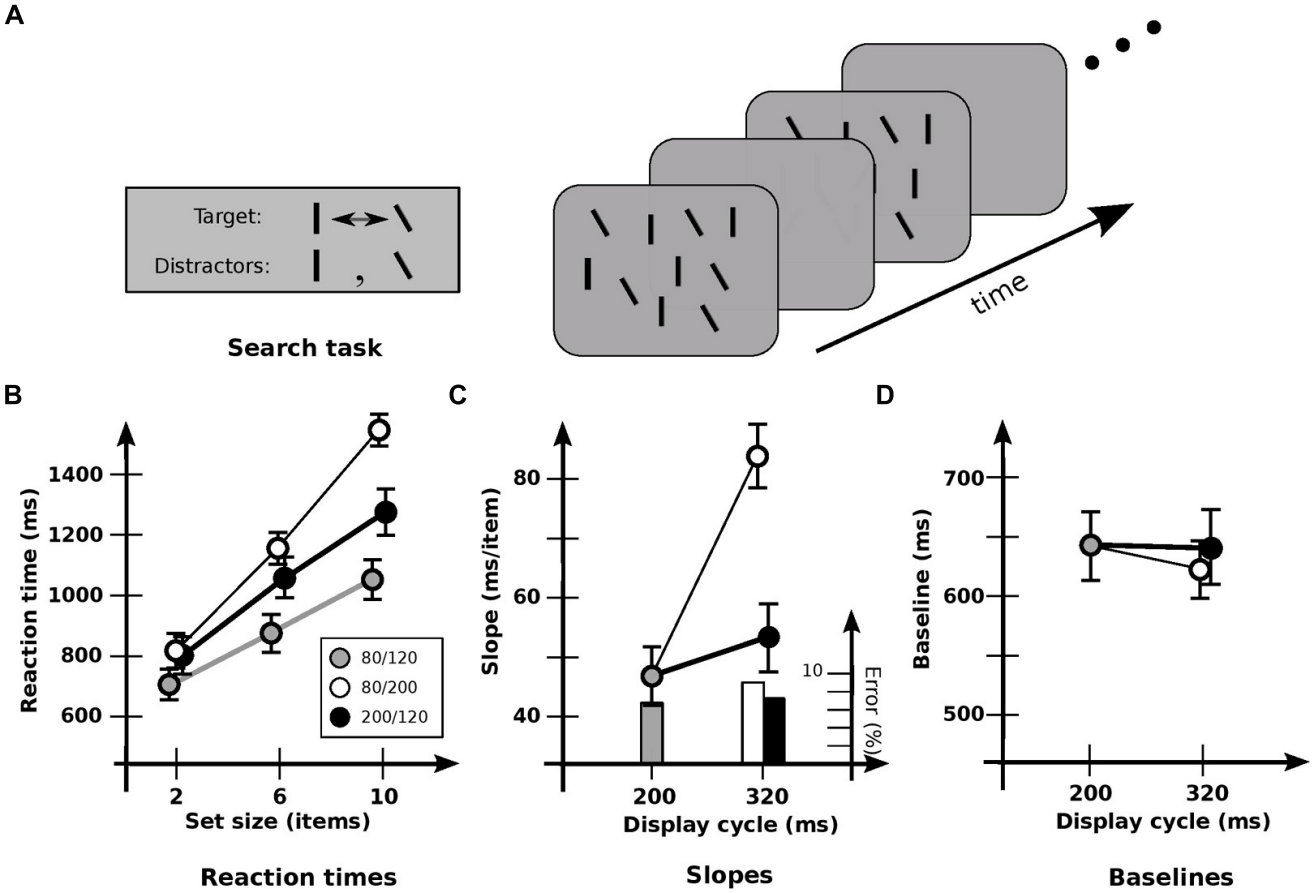
**Experiment 2: detection of orientation change. (A)** Stimuli used. ~50% of lines in each display are vertical, and 50% are tilted by 30° counterclockwise. Target is the item that changes between vertical and tilted; distractors are those items that maintain a constant orientation. **(B)** Response times and error rates as a function of set size for the three cadences. **(C)** Data recast as slopes. Slope for base cadence (47.3 ms/item) is strongly affected by an increase in off-time (83.0 ms/item) but not by an increase in on-time (53.7 ms/item). **(D)** Data recast as baselines. Values for the 80/240 and 200/120 cadences have been subtracted by 120 ms to equate the time of first appearance of the changed item. Baseline for base cadence (645 ms) is not significantly affected by an increase in off-time (615 ms) or on-time (641 ms). Error bars indicate standard error of the mean.

Experiment 2 used the same set sizes and much the same items as in Experiment 1A. The same cadences were also used, so that any interference from the flickering displays would be about the same. However, each display now contained approximately equal numbers of vertical lines and lines tilted counterclockwise by 30°. The target was now the item that *changed* its orientation by 30° between displays (**Figure 2A**).

As before, set size had a strong effect on RT [*F*(2,11) = 172.1; *p* < 0.0001; **Figure 2B**]. But there was now a significant effect of cadence [*F*(2,11) = 27.4; *p* < 0.0001] and a significant interaction between set size and cadence [*F*(4,11) = 24.5; *p* < 0.0001]. In particular, cadence had a strong effect on slopes [*F*(2,11) = 33.0; *p* < 0.0001; **Figure 2C**], which were higher with increased off-time (*p* < 0.001). However, there was no effect with increased on-time (*p* > 0.2), again indicating that the different rates of switching between visual and iconic representations had little effect. Baselines (**Figure 2D**) were not reliably affected [*F*(2,11) = 1.31; *p* > 0.2). (In general, baselines were never reliably different in all the conditions that follow, and so are omitted from subsequent analyses.)

Interpreting slopes in terms of the number of items held across the blank interval (Rensink, 2000b), a strong effect of cadence was again evident [*F*(2,11) = 20.1; *p* < 0.0001]. However, the opposite pattern now occurred: hold did not differ significantly with greater off-time (*p* > 0.05), but increased with greater *on-time* (*p* < 0.005). This is consistent with the proposal that under these conditions the speed of change detection is largely governed by the loading of information into visual short-term memory (vSTM) and its subsequent comparison (Rensink, 2000b). It also suggests that these operations take place largely during on-times alone, being largely unable to use iconic memory. Indeed, a more detailed analysis of the slopes shows that performance is a function of on-time plus a usable duration of *u* = 115 ± 18 ms. [Note that if usable duration started from stimulus *onset*, the similar speeds for the 80/120 and 200/120 cadences would require a value of at least 320 ms. But then there would be similar speeds for the 80/240 and 200/120 cadences, which was not the case (*p* < 0.0001). Thus, usable duration apparently begins at stimulus *offset*.]

For the detection of both orientation and contrast changes, the loading of information into vSTM is proportional to the duration of the display plus ~110 ms (Rensink, 2000b, Figure 6). Since the ISI in those conditions was 120 ms, this indicates that usable duration *u* is not the “worth” of iconic memory (Loftus et al., 1992), but an actual time limit. Once this limit is exceeded, iconic memory simply cannot be used for change detection, even though the results of Experiment 1 indicate that it still exists, and contains potentially usable information.

## EXPERIMENT 3

**FIGURE 3:**
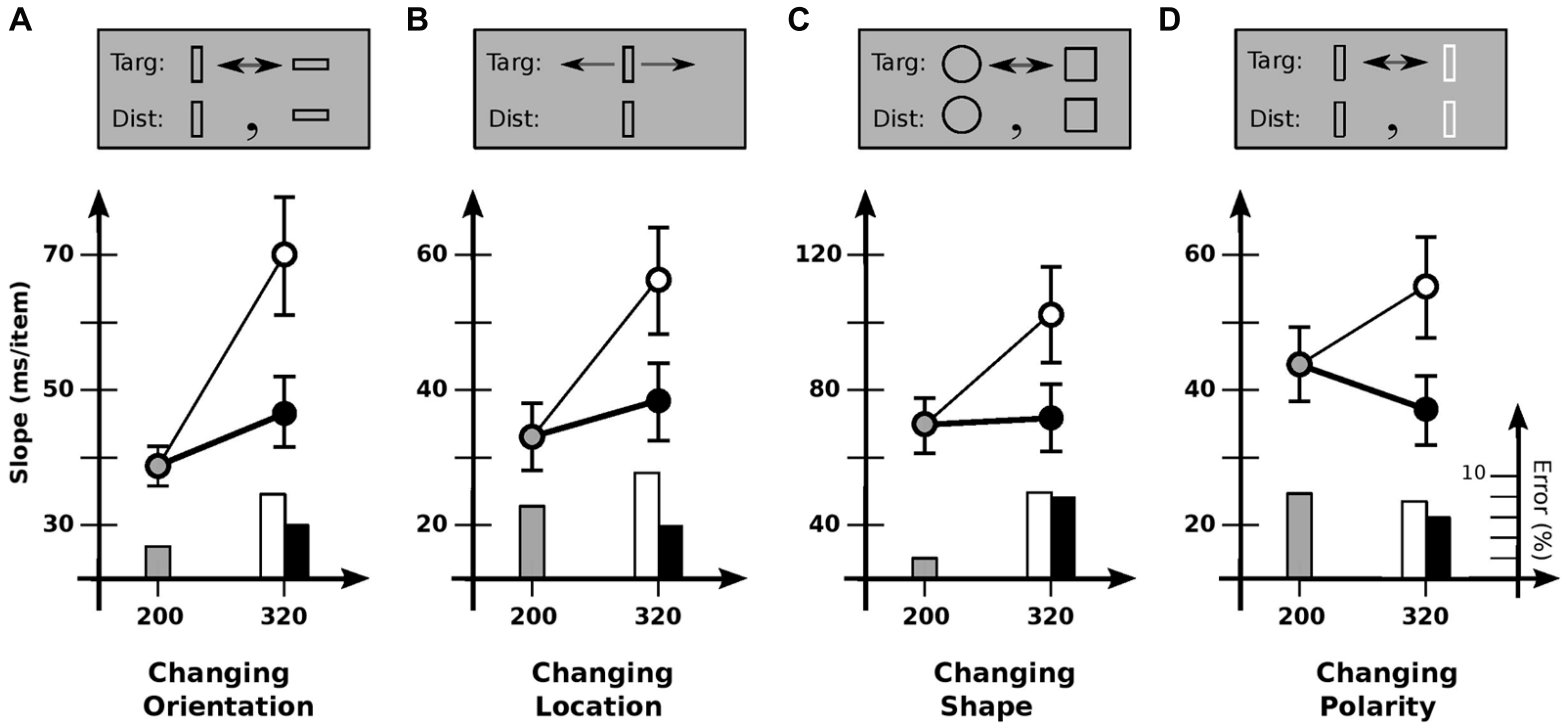
**Experiment 3: detection of different kinds of feature change. (A)** Changing orientation. Slope for base cadence (38.7 ms/item) is strongly affected by an increase in off-time (69.7 ms/item) but not an increase in on-time (47.0 ms/item). **(B)** Changing location. Slope for base cadence (33.1 ms/item) is strongly affected by an increase in off-time (56.1 ms/item) but not an increase in on-time (38.0 ms/item). **(C)** Changing shape. Slope for base cadence (69.4 ms/item) is strongly affected by an increase in off-time (101.9 ms/item) but not an increase in on-time (70.2 ms/item). **(D)** Changing polarity. Slope for the base cadence (43.2 ms/item) is significantly affected by an increase in off-time (55.7 ms/item), but marginally affected by an increase in on-time (37.6 ms/item). Error bars indicate standard error of the mean.

To explore the generality of the limited usability found in Experiment 2, Experiment 3 investigated other kinds of items and kinds of change (**Figure 3**). Conditions were otherwise much the same. In Condition 3A, items were rectangular outlines 0.4° × 1.2°, with targets changing orientation 90° between vertical and horizontal (**Figure 3A**). As in Experiment 2, slopes depended strongly on cadence [*F*(2,11) = 14.4; *p* < 0.0001], with search slowing reliably for increased off-time (*p* < 0.001) but not increased on-time (*p* > 0.05). Usable duration *u* was 117 ± 27 ms, much the same as before.

Condition 3B examined change in location. Here, the target jumped back and forth 1.2° each alternation, with distractors remaining stationary. Slopes again depended on cadence [*F*(2,10) = 12.7; *p* < 0.0002], with search slowing for increased off-time (*p* < 0.0002) but not increased on-time (*p* > 0.3). Usable duration *u* was 123 ± 34 ms, similar to previous values.

Condition 3C looked at shape change, with the target alternating between a circle and a square. Although more difficult than the other conditions, similar results were found: slope depended on cadence [*F*(2,11) = 9.9; *p* < 0.001], with search slowing for increased off-time (*p* < 0.01) but not increased on-time (*p* > 0.9). Usable duration *u* was 139 ± 37 ms, comparable to previous values.

Finally, condition 3D examined changes in contrast polarity (black vs. white). Slopes again depended on cadence [*F*(2,11) = 8.8; *p* < 0.002]. Search slowed down with increased off-time (*p* < 0.05), and tended to speed up with increased on-time, although statistical reliability was marginal (*p* = 0.06). [This latter effect has been found elsewhere, where it was taken to indicate a grouping process—based on polarity—that takes place over several 100 ms (Rensink, 2000b).] Comparing the 80/240 and 200/120 cadences (which equates time per alternation) shows search to be reliably faster with greater on-time (*p* < 0.005); relative speeds yield *u*= 137 ± 34 ms, similar to the values for other kinds of change. In summary, then, all change detection tasks appeared to show the same kind of behavior, with the same usable duration of about 120 ms.

## EXPERIMENT 4

**FIGURE 4:**
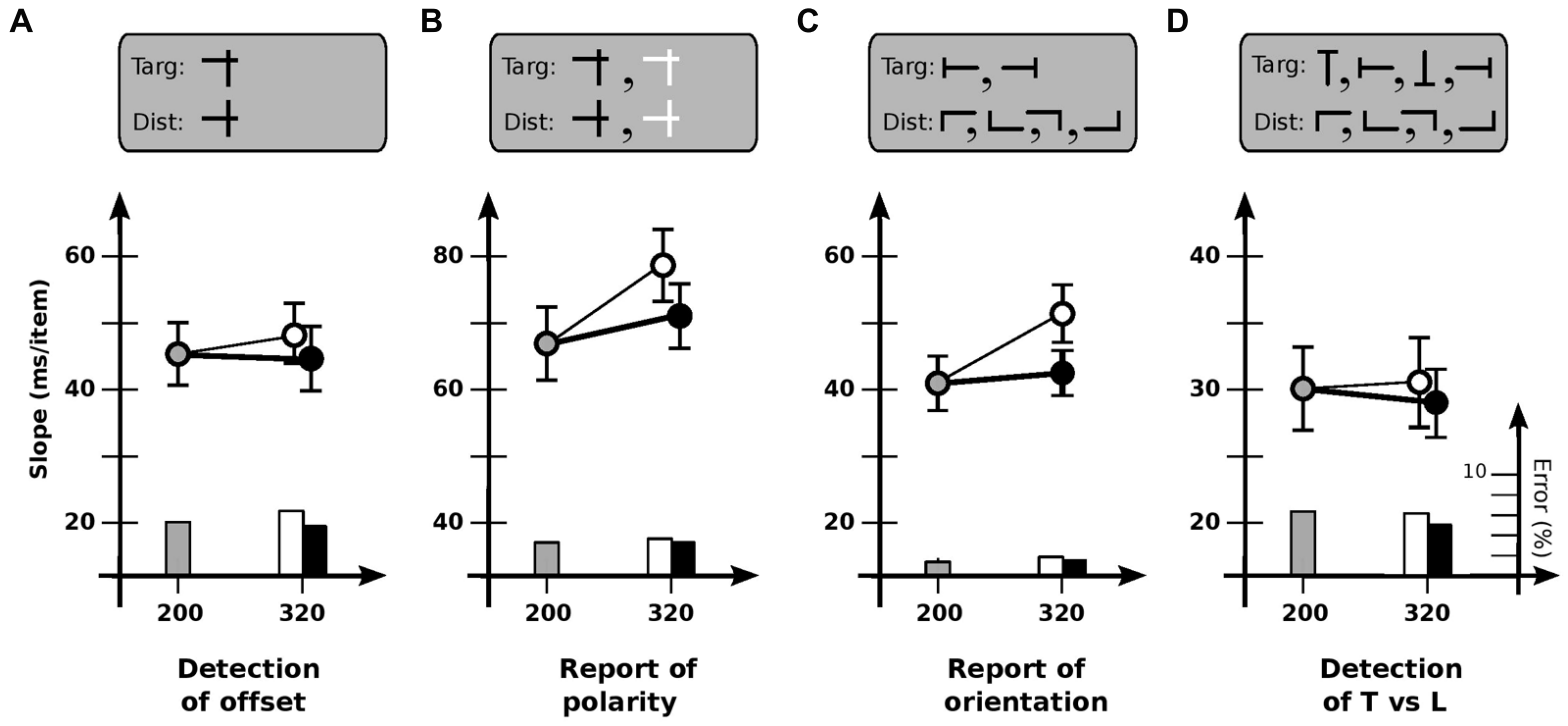
**Experiment 4: different tasks. (A)** Detection of offset horizontal line. Slope for base cadence (45.6 ms/item) is unaffected by an increase in off-time (47.6 ms/item) or on-time (44.7 ms/item). **(B)** Report of contrast polarity of offset horizontal line. Slope for base cadence (66.6 ms/item) is reliably affected by an increase in off-time (78.0 ms/item) but not an increase in on-time (71.1 ms/item). **(C)** Report of orientation of T-shaped item. Slope for base cadence (40.8 ms/item) is affected by an increase in off-time (50.7 ms/item) but not an increase in on-time (42.0 ms/item). **(D)** Detection of T-shaped item. Slope for base cadence (30.0 ms/item) is unaffected by an increase in off-time (30.5 ms/item) or on-time (28.2 ms/item). Error bars indicate standard error of the mean.

Experiment 4 investigated why the usability of iconic memory might be limited for some tasks but not others. To determine if task difficulty was important, Condition 4A gave observers a simple detection task (as in Experiment 1), with the target defined by a horizontal bar only slightly higher than those of the distractors. Speeds were now comparable to several of those in Experiments 2 and 3 (**Figure 4A**). However, cadence did not have much of an effect [*F*(2,11) = 0.28; *p* > 0.7], indicating that difficulty *per se* was not the critical factor.

To determine if usable duration might be different if a report is required of the target, Condition 4B used much the same items as in Condition 4A, but with half being black and half white; observers were asked to *identify* the contrast of the target rather than detect it (**Figure 4B**). Dependence on cadence now reappeared [*F*(2,11) = 4.0; *p* < 0.05], with search slowing for increased off-time (*p* < 0.01) but not increased on-time (*p* > 0.3). Usable duration *u* was 202 ± 29 ms, less than the 240 ms (or higher) limit of a static detection task, but greater than the values for a change detection task.

To determine if this value might have somehow been due to the mixed polarity of the items, Condition 4C tested report of the orientation of a T-shaped target (left or right) among L-shaped distractors; all items were black (**Figure 4C**). Search again depended on cadence [*F*(2,11) = 10.3; *p* < 0.001], slowing for increased off-time (*p* < 0.002) but not increased on-time (*p* > 0.5). Usable duration *u* was 181 ± 26 ms, similar to that for Condition 4B.

Finally, to examine whether the key factor in Conditions 4B and 4C might have been the existence of multiple kinds of target, Condition 4D asked observers to detect (but not report on) a T-shaped target among L-shaped distractors, with all items—targets as well as distractors—in any of four orientations (**Figure 4D**). Dependence on cadence now vanished [*F*(2,11) = 0.49; *p* > 0.6], indicating that multiplicity was not important.

Taken together, then, the results above suggest that the critical factor determining the extent to which iconic memory can be used is not the difficulty of the task or the kinds of items involved, but something about the task itself. A common element of change detection and report—but not static detection—is the need for an item to be *individuated*, i.e., treated as a particular individual at a particular location (Smith, 1998; Pylyshyn, 2003). In change detection, for example, an item that is initially seen (and stored in vSTM) must be re-identified as the same item in the subsequent presentation. Likewise in report, an item detected on the basis of some given feature must be identified as such by whatever process underlies the subsequent report. Such individuated items are believed to play a key role in many visual processes (Ullman, 1984).

## GENERAL DISCUSSION

The results above indicate that for all visual search tasks, iconic memory can act as a surrogate for about 120 ms: during this time it can be used as easily and effectively as if the original stimulus were present. Results also show that for some—but not all—tasks, it is available for much longer. The key factor is not the difficulty of the task or the type of feature involved; instead, it appears to be the extent to which the task relies on individuation. Three groups of limits were encountered: for change detection, ~120 ms; for report, 190 ms; for static detection, at least 240 ms. The existence of these groups suggests that iconic memory is not a monolithic structure, but involves several (spatially organized) layers, drawn upon by different tasks to different extents.

Traditionally, iconic memory is taken as having two components: the first a high-density, retinotopic *visible persistence* existing up to 200 ms from stimulus onset (exact value depending on lighting level), and the second a longer-lasting *informational persistence* that is more abstract and mediated more centrally (Coltheart, 1980; Loftus and Irwin, 1998). Since visible persistence can last on the order of a 100 ms under some conditions (Coltheart, 1980), it may be part of the fastest-decaying layer. However, access to the other layers lasts much longer; as such, they would likely involve only informational persistence.

What might these layers correspond to? One possibility involves re-entrant connections from higher level visual areas to lower level ones. Complex static patterns can be detected by neurons in areas such as temporal cortex; cells here have a considerable degree of spatial invariance, responding to much of the visual field (e.g., Felleman and Van Essen, 1991). But to individuate an item—to see it as a particular individual at a particular location—requires linking these spatially invariant representations to lower level retinotopic ones. This can be done, for example, by correlating downward, spatially diffuse signals from higher levels with upward, spatially precise ones from striate cortex (Di Lollo et al., 2000; Tsotsos, 2011).

Results from several lines of research are consistent with this general view. Massive numbers of re-entrant connections exist between the cortical areas involved in visual perception (e.g., Felleman and Van Essen, 1991; Bullier, 2004). Such connections can explain phenomena such as common-onset masking (Di Lollo et al., 2000) and context effects in recognition (Weisstein and Harris, 1974); indeed, they are believed to be involved in a large variety of visual processes (Fukushima et al., 1991; Tsotsos, 2011). As such, the representation of an item—a *visual object*—is distributed over several levels, with its representation at these levels “knit” together by feedforward and feedback circuits (e.g., Rensink, 2000a, 2002).

Looked at in this way, the different layers of iconic memory could correspond to the memory traces at these different levels (cf. Keysers et al., 2005; Ruff et al., 2007). After a stimulus disappears, representations at the various levels—or at least, their connections—begin to decay, with different time constants at each level. Given that durations are generally longer at higher visual areas (Keysers et al., 2005), the more detailed representations at lower levels would likely be the first to go. If so, the layer accessible for only 120 ms would likely correspond to the lower level representations. (Visible persistence may be part of this.) Given that this layer is needed for change detection, it would likely contain relatively precise spatial information, needed to ensure continuity of representation over time (Rensink, 2000a, 2013). Meanwhile, layers that are usable for longer durations might reflect higher level representations, which are more abstract and have poorer spatial localization.

Such as *multi-layer* theory of iconic memory could explain the usable durations for the different kinds of task as follows:

(a) *Static detection (≥240 ms).* Information carried by the feedforward “wave” created by the appearance of an item reaches high levels relatively quickly. After a brief time (c. 100 ms), access to high-precision spatial information in the low iconic layers begins to degrade. But since detection does not require precise spatial information, it can still be “driven” by the information at the higher layers of iconic memory for several 100 ms longer. This can explain many classic partial report results, which require only a report of a stimulus (generally, a letter) at some coarsely specified location, but not its precise position. Note that although absolute position is eventually lost at higher levels, precise *relative* positions could still be maintained. For example, the targets in Condition 4A differed from the distractors by only a small shift in the position of a horizontal bar; this information remained available for at least 240 ms. Consistent with this, partial report studies suggest that shape information in iconic memory can remain fairly accurate for over 300 ms (Gegenfurtner and Sperling, 1993; Graziano and Sigman, 2008).
(b) *Change detection (c. 120 ms).* The relatively short usable duration (120 ms) for change detection could reflect the need for precise spatial location, which is required for item continuity (Rensink, 2000a, 2013). An important issue is whether this duration reflects the decay of the *contents* of the low-level representation, or just the *connections* to it. Studies based on exogenous cues indicate that positional information does not degrade greatly for at least 300 ms (Graziano and Sigman, 2008). And since exogenous cues can make use of—and transmit—the location of these cues, it would appear that feedforward connections can be maintained, at least for spatial information of moderate resolution. In contrast, the process of establishing a feedback connection to lower levels needs spatial information that is very precise (Di Lollo et al., 2000); such connections might therefore fail relatively quickly.
(c) *Report (c. 190 ms).* For the report tasks of Conditions 4B and 4C, usable duration is greater than that for change detection but less than that for static detection. At least two explanations are possible. First, it may be that detection proceeds as usual, but a subsequent individuation stage is needed to report the associated properties of the detected item; usable duration would then reflect the relative amount of time needed for each of these stages. Consistent with this, slopes of the report tasks were 10–20 ms/item greater than those of their detection equivalents (**Figure 4**), suggesting the involvement of an additional processing stage. Alternatively, individuation may only need to be partial—i.e., the representation of the target item need only be linked back to a level where its location can be readily distinguished from those of the others. If so, feedback connections may only be established with a mid-level layer, which may endure somewhat longer than those at lower levels.

### Relation to other work

Among other things, the proposal here is consistent with results on attentional capture and apparent motion that show a visual continuity for 100 ms after the disappearance of an item (e.g., Yantis and Gibson, 1994). It is also consistent with findings of partial report experiments that (i) when a mask is shown after stimulus disappearance, identification errors arise only if the mask is shown within 150 ms or so of stimulus onset, while localization errors can be induced even if the mask is presented much later, and (ii) if a mask is not used, localization errors begin soon after stimulus disappearance, while identification errors remain low (Mewhort et al., 1981). These patterns can be explained by the existence of a durable array (or “buffer”) of fairly complex but poorly localized information at higher levels, along with a relatively fast decay of their connections to spatial locations at lower ones.

The proposal of multiple layers of iconic memory is also similar in some ways to the proposal of multiple systems of visual memory (e.g., Sligte et al., 2010). There is general agreement with the idea of detailed, volatile representations at the lower levels, along with a single detailed, longer-lasting representation (corresponding to a visual object) held in vSTM (cf. Rensink, 2000a, 2002). Multiple-systems experiments are based on the use of positional cues with delays of several seconds. Since this is beyond the lifetime of “classic” iconic memory, they are likely concerned with longer-lasting—and likely more limited—representations. The exact nature of this memory is not completely understood; indeed, the existence of a distinct “fragile” vSTM is still controversial (see e.g., Makovski, 2012). But if multiple systems do exist, they could be higher level counterparts of the layers proposed here.

### Iconic memory, feedback connections, and visual attention

The theory of iconic memory described here also has implications for the role of feedback processes in human vision. Anatomical and physiological studies indicate that human vision relies upon two main types of feedback connections (e.g., Bullier, 2004). The first are *horizontal* connections of adjacent cells at the same level of the processing stream; these converge quickly and can potentially support rapid local computation of considerable complexity, such as determination of local shape. Given the durability of high-accuracy (local) shape representation in iconic memory (Mewhort et al., 1981), such connections appear to be relatively long-lasting. Longer-range connections can also exist between corresponding locations in representations at the same level of the visual hierarchy (e.g., representations of color and orientation). The second type of connection involves *vertical* links between corresponding cells at different levels. As discussed above, the memory at each of these levels—and in the connections between them—may be the basis of the iconic layers proposed here.

It has been proposed that “the representation of any item in this form of storage [iconic memory] is achieved by creating a temporary file of information about the item” (Coltheart, 1983, p. 291), with relatively complex structure (such as characters) created in parallel across the visual field, but susceptible to overwriting by the subsequent appearance of other structures (Mewhort et al., 1981; Coltheart, 1983). This is similar to the proposal of *proto-objects* (Rensink and Enns, 1998), which are relatively complex structures of limited extent formed rapidly and in parallel in the (near-) absence of attention; these too are temporary, either fading away within a few 100 ms, or being overwritten by the representation of a new item that appears at their location (Rensink et al., 1997). Fast-acting horizontal connections could explain why the within-item binding needed for proto-objects can be achieved using so little time and so little attention. They could also explain why considerable binding exists in iconic memory (Landman et al., 2003), even in its lowest layers (Experiments 1 and 4A).

Meanwhile, vertical connections could be the basis of larger-scale representations. Feedforward and feedback links likely connect corresponding locations at different levels in a fairly dense way (e.g., Di Lollo et al., 2000). Such links could enable retinotopic representations at low-levels (e.g., in striate cortex) to connect to spatiotopic representations at high ones (e.g., in temporal cortex) via a series of stages in which position is increasingly less tied to retinal location (e.g., Tsotsos, 2011). And attention might act by establishing long-range feedforward-feedback loops to represent a coherent visual object, resulting in a representation distributed across the various levels, their contents linked via circuits connecting contents at the same (relative) spatial location (Rensink, 2000a, 2002; Lamme, 2003; Sligte et al., 2010; Tsotsos, 2011).

Characterizing “iconic,” “preattentive,” and “attentive” representations in this way can account for why performance on iconic and visible representations is so similar (Experiment 1), why selective attention and readout from iconic memory involve common neural mechanisms (e.g., Ruff et al., 2007), and why there is little cost for switching between the two (Experiments 1 and 4A). Said simply, there is no separate “iconic” memory system: the layers of iconic memory are just the traces of the representations through which normal visual perception proceeds (see also Keysers et al., 2005; Ruff et al., 2007).

In this view, iconic memory—or at least, informational persistence—has a clear purpose: *to help establish and maintain links between the various spatially organized representations of an item.* Given the decreasing precision of representations with increasing level, processes based on a feedforward sweep of information could continue to use such information even after the contents at the lower levels have faded. However, processes relying on feedback from higher levels would not always have access to the more detailed (but volatile) representations at lower ones; when this happens, the process must wait for the contents of these to be re-instantiated.

The extent to which this proposal adequately captures the operation of the visual system is unclear. But to the degree that it is relevant, the “usability logic” developed here could provide a useful way to investigate the various feedforward and feedback mechanisms involved.

## AUTHOR CONTRIBUTIONS

Much of this work was done while the author was with Cambridge Basic Research, Nissan Research & Development, Inc., Cambridge, MA, USA. Portions were presented at the annual conference of the Association for Research in Vision and Ophthalmology in May 1997, and the European Conference on Visual Perception in August 2008. Many thanks to Duncan Bryce, Emily Cramer, Puishan Lam, Kyle Melnick, Nayantara Santhi, and Monica Strauss for their help in running the experiments.

## Conflict of Interest Statement

The author declares that the research was conducted in the absence of any commercial or financial relationships that could be construed as a potential conflict of interest.

## Footnotes

1 If the start of search after display onset is stochastic, and the variance of this is sufficient, random sampling will ensure that the fraction of on- or off-time encountered will on average be that in the display cycle. To help with this, observers were dropped from the analysis if search was over before the first display cycle was complete—i.e., before a full testing of the first iconic representation could be made. The criterion used was that search should be slow enough to allow the complete testing of 10 items (the maximum present) duration a single display cycle at the slowest cadence (320 ms). Note that this does not assume an item-by-item scan of the display; attention could be allocated to the items in parallel. On the basis of this criterion, two observers were removed: one from Experiment 1A, and one from Experiment 3C. More severe criteria did not significantly change the overall pattern of results. For even the fastest search encountered here (c. 50 ms/item in Experiment 1A), a scan of both visual and iconic representations was essentially complete for displays containing only six items. Importantly, cadence affected only the slopes and not the shapes of the response-time curves (Figures 1 and 4). This provides evidence that the timing assumptions underlying this technique are reasonably accurate for the conditions examined here.

2 The term “re-entry” generally denotes a particular type of feedback, viz., that in which density of back connections is similar to or exceeds the density of forward connections, and for which the mapping of back connections is not haphazard, but has a mapping similar to that of the feedforward connections. In the context here, “re-entry” and “feedback” will be considered synonymous.

3 Slopes for each observer were calculated by determining mean response time for each set size, and calculating a least-squares fit through these points. Analysis used repeated-measures ANOVAs, and paired, two-tailed *t*-tests. Target-present slopes were used; target-absent slopes either followed the same pattern or showed no strong effects. Error rates in the target-absent condition were generally low (below 2%) and did not vary much over different conditions. Errors for target-present conditions either followed the pattern of the slopes or showed no strong effects, indicating that speed-accuracy trade-off was not a factor.

4 Usable memory duration *u* can be calculated in the following way. The total usable time in each alteration is taken to be the duration of the visible component plus the usable duration of the iconic component. Assuming the usable duration in the 80/120 and 200/120 cadences is 120 ms or more, and that speed is the same for visible and iconic inputs (both assumptions supported by the results of Experiment 1), search speed can be estimated by averaging the slopes of the two short-ISI cadences to get slope s_V_, corresponding to search through a visible representation. The usable fraction *f* over a complete display cycle is s_V_/s_L_, where s_L_ is the slope of the long-ISI cadence. For a long-ISI condition with on-time of 80 ms and display cycle ( = on-time + off-time) of D ms, *f* is also *(80+u)/D*; rewriting, *u = Df – 80*= *D*(s_V_/s_L_) – 80. The standard error of the mean of *u* can be determined from this formula, via the standard errors of the slopes.
